# Tooth development, histology, and enamel microstructure in *Changchunsaurus parvus*: Implications for dental evolution in ornithopod dinosaurs

**DOI:** 10.1371/journal.pone.0205206

**Published:** 2018-11-07

**Authors:** Jun Chen, Aaron R. H. LeBlanc, Liyong Jin, Timothy Huang, Robert R. Reisz

**Affiliations:** 1 International Center of Future Science, Dinosaur Evolution Research Center, Jilin University, Changchun, China; 2 The Key-Lab for Evolution of Past Life and Environment in Northeast Asia, Ministry of Education, China, Changchun, China; 3 Department of Biological Sciences, University of Alberta, Edmonton, Alberta, Canada; 4 Department of Biology, University of Toronto Mississauga, Mississauga, Ontario, Canada; Ecole Normale Supérieure de Lyon, FRANCE

## Abstract

The great diversity of dinosaurian tooth shapes and sizes, and in particular, the amazing dental complexity in derived ornithischians has attracted a lot of attention. However, the evolution of dental batteries in hadrosaurids and ceratopsids is difficult to understand without a broader comparative framework. Here we describe tooth histology and development in the "middle" Cretaceous ornithischian dinosaur *Changchunsaurus parvus*, a small herbivore that has been characterized as an early ornithopod, or even as a more basal ornithischian. We use this taxon to show how a "typical" ornithischian dentition develops, copes with wear, and undergoes tooth replacement. Although in most respects the histological properties of their teeth are similar to those of other dinosaurs, we show that, as in other more derived ornithischians, in *C*. *parvus* the pulp chamber is not invaded fully by the newly developing replacement tooth until eruption is nearly complete. This allowed *C*. *parvus* to maintain an uninterrupted shearing surface along a single tooth row, while undergoing continuous tooth replacement. Our histological sections also show that the replacement foramina on the lingual surfaces of the jaws are likely the entry points for an externally placed dental lamina, a feature found in many other ornithischian dinosaurs. Surprisingly, our histological analysis also revealed the presence of wavy enamel, the phylogenetically earliest occurrence of this type of tissue. This contradicts previous interpretations that this peculiar type of enamel arose in association with more complex hadrosauroid dentitions. In view of its early appearance, we suggest that wavy enamel may have evolved in association with a shearing-type dentition in a roughly symmetrically-enameled crown, although its precise function still remains somewhat of a mystery.

## Introduction

Comparative dental histology is an emerging area of study in dinosaur palaeontology [1–18] and the breadth and depth of this research is increasing. Several studies have focused on the hard tissues -enamel and dentine—and their phylogenetic and functional significance [4,19–22], but the field has steadily broadened to include more holistic descriptions and comparisons of teeth, their constituent tissues, and their interactions with the jaws. The result has been a broader picture of how teeth and jaws have evolved in association with dietary shifts and what developmental processes could have given rise to novel dentitions [2,5,7–9,13,14,17,23–26]. Some of the most dramatic and most studied evolutionary innovations are found in derived ornithischian dinosaurs.

Ornithischian dinosaurs display an amazing diversity of tooth shapes, sizes, and arrangements to cope with their herbivorous diets [2,9,10,13,27–31]. The teeth of most early ornithischian dinosaurs were relatively simple triangular or leaf-shaped teeth for shearing plant material [32], but hadrosaurid and ceratopsid dinosaurs displayed extensive modifications to their dentitions, forming complex dental batteries for more efficient oral processing. The evolution of these complex structures, particularly the dental batteries of hadrosaurids, has been studied extensively and figures prominently in many histological analyses of dinosaur teeth [1–4,6,9,16,22,33]. The appeal of understanding how such complex structures originated and functioned is obvious, however without a broader comparative framework, it is difficult to characterize the evolutionary history of dental complexity in derived dinosaurian clades [5]. Whereas several studies have revealed important insights into dental anatomy and replacement in phylogenetically earlier ornithischians [34–38] our understanding of dental histology and development in many of these ornithischian clades remains poor compared to the wealth of research efforts focused on more specialized, dental battery-bearing taxa.

Here we describe tooth histology and development in several jaws of the Cretaceous (Aptian–Cenomanian) ornithischian dinosaur, *Changchunsaurus parvus* [39] from the Quantou Formation of Jilin Province, China. This taxon has been pivotal to our understanding of ornithischian evolution, because of its potential placement as an early ornithopod [40], or as an earlier member of Ornithischia [41]. The contentious placement of this taxon makes it ideally suited to understand (1) how a more “typical” ornithischian dentition develops, copes with tooth wear, and is maintained through ontogeny (via tooth replacement); and (2) if phylogenetically earlier ornithischians show dental adaptations that are similar to those present in the complex dental batteries of hadrosaurids, in particular. Moreover, given the growing comparative histological dataset of dinosaur dentitions, we can compare these aspects of tooth development and histology in *C*. *parvus* to those of other herbivorous and carnivorous dinosaurs in order to identify unique aspects of the dentition in this ornithischian dinosaur.

## Materials and methods

We examined five partial dentaries from different individuals of *Changchunsaurus parvus*, from Jilin University Museum collections (JLUM; Table 1), and carried out the histological section experiments at the ROM vertebrate palaeohistology laboratory.

**Table 1:** Specimens of *Changchunsaurus parvus* sectioned in this study.

Specimen	Section	Specimen nature and catalogue number	ROM Thin Section number
1	1	Maxilla fragment Ya-C160711e1	TS01030
	2	Maxilla fragment Ya-C160711e1	TS01031
2	3	Dentary fragment Ya-C160711e2	TS01032
	4	Dentary fragment Ya-C160711e2	TS01033
3	5	Dentary fragment Ya-C160711e3	TS01034
	6	Dentary fragment Ya-C160711e3	TS01035
4	7	Dentary-left Ya-C160711a	TS01042
	8	Dentary-left Ya-C160711a	TS01043
5	9	Dentary-right Ya-C160711b	TS01044
	10	Dentary-right Ya-C160711b	TS01045
6	11	Dentary-right Ya-C160711c	TS01046
	12	Dentary-right Ya-C160711c	TS01047
	13	Dentary-right Ya-C160711c	TS01054
7	14	Dentary-left Ya-C160711d	TS01048
	15	Dentary-left Ya-C160711d	TS01049

The histological thin sections of several partial *C*. *parvus* dentaries (Table 1) were prepared by first embedding specimens in Castolite AP polyester resin and placing them under vacuum. Embedded materials were then cut using a Buehler Isomet 1000 wafer blade saw set to 200–300 rpm to ensure a smooth cut. The cut surfaces were then polished using 600-grit and 1000-grit silicon carbide powder. Specimens were later mounted to frosted plexiglass slides using cyanoacrylate and a small waver was cut again using the Isomet saw. The mounted waver was then ground down using a Hillquist grinding machine and further polished using progressively finer grits of silicon carbide and aluminium oxide powders. The thin sections were imaged using a Nikon DS-Fi camera mounted to a Nikon AZ-100 microscope with NIS Elements BR imaging software registered to D. C. Evans or R. R. Reisz, and Leica DVM6 Transmitting Scope and Tilting Scope, and SEM registered to R. R. Reisz.

Some histological slides were acid-etched and imaged using a JEOL NeoScope JCM-5000 Scanning Electron Microscope (SEM) to examine the enamel microstructure. These specimens were not coated or treated prior to being placed in the SEM. The terms used in describing reptilian enamel microstructure are thoroughly explained in Sander [22]. Terms specific to dinosaur enamel microstructure are detailed in Hwang [4].

## Results

### Tooth development and replacement cycle

**Fig 1:**
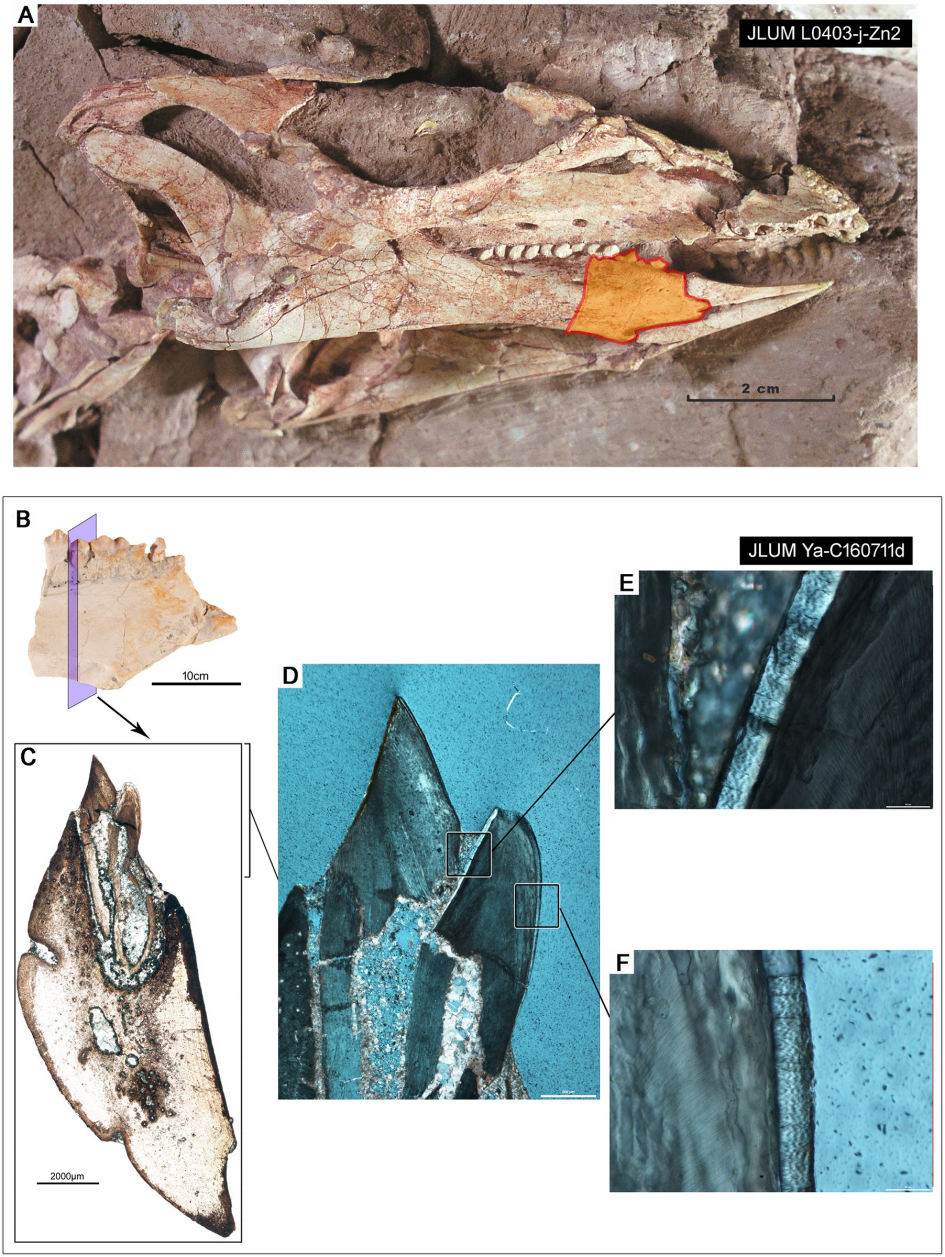
The skull and thin sections of the jaws of *Changchunsaurus parvus*. **A.** Image of the skull of the holotype specimen of *C*. *parvus* (JLUM L0304-j-Zn2). **B.** Image of one of the partial dentaries sampled in this study, with the plane of section highlighted (JLUM Ya-C160711d). **C.** Whole-view image of one thin section (TSO1049) through the dentary showing a functional and nearly erupted replacement tooth. **D.** Close-up, cross-polarized image of the tooth crowns of the functional and replacement teeth in C. **E.** Close-up, cross-polarized image of the wavy enamel along the labial surface of the replacement tooth crown. **F.** Close-up, cross-polarized image of the wavy enamel along the lingual margin of the replacement tooth crown.

**Fig 2:**
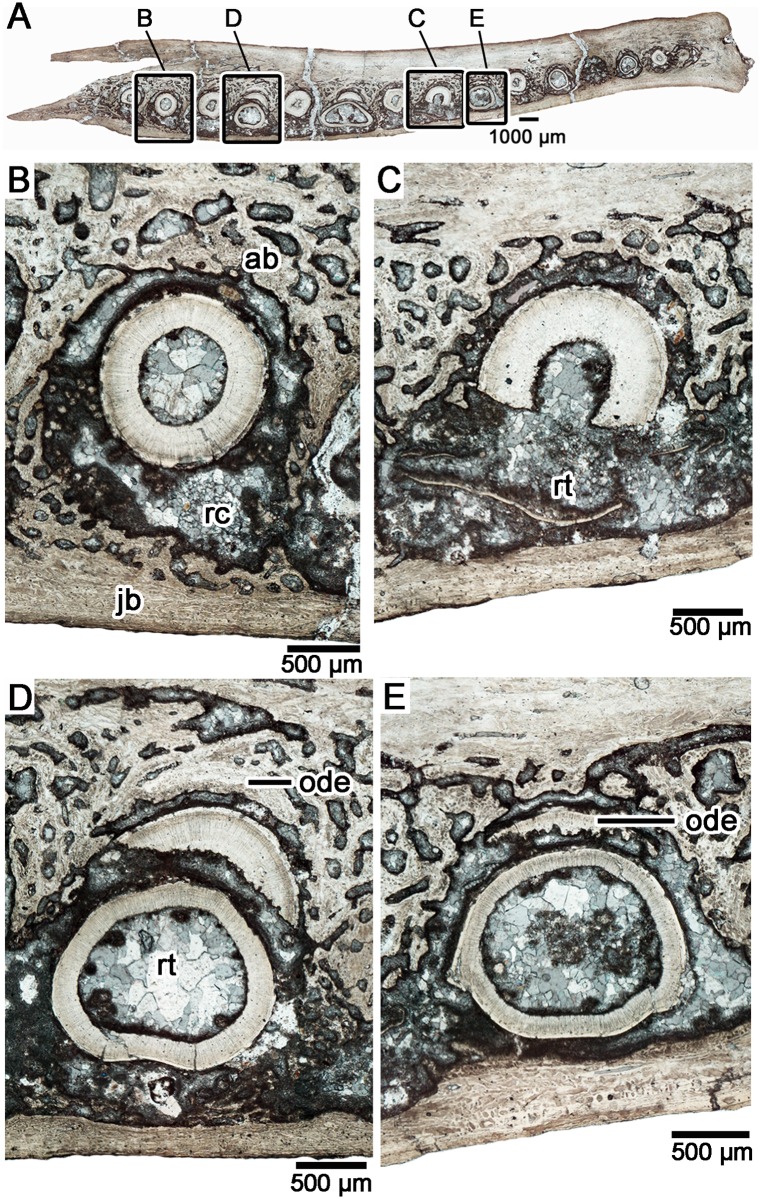
Tooth histology and a reconstruction of the replacement cycle in a transverse section of the dentary teeth in *Changchunsaurus parvus*. (JLUM-Ya-C160711C). **A.** Whole-view of a transverse section (TS01054) across the dentary tooth series. Anterior is to the right, and lingual towards the bottom of the image. **B.** Close-up image of a tooth position showing the early stages of replacement. The hard tissues of the replacement tooth are not visible and only a small replacement crypt is present lingual to the functional tooth. **C.** A tooth position showing the development of the large, labiolingually compressed tooth crown lingual to the functional tooth. Some root resorption has already occurred in the functional tooth. **D.** A tooth position showing an advanced stage of tooth replacement. The replacement tooth is migrating towards the occlusal margin and the majority of the functional tooth root has been resorbed. Note the presence of a remnant of dentine from a previous tooth generation embedded in the labial wall of the alveolus. **E.** A tooth position showing the latest stage of tooth replacement. The functional tooth has been shed, leaving behind a small remnant of the tooth root at the labial margin of the alveolus. **Abbreviations: ab,** alveolar bone; **jb,** bone of the jaw; **ode,** old remnant of dentine; **rc,** resorption crypt; **rt,** replacement tooth.

**Fig 3:**
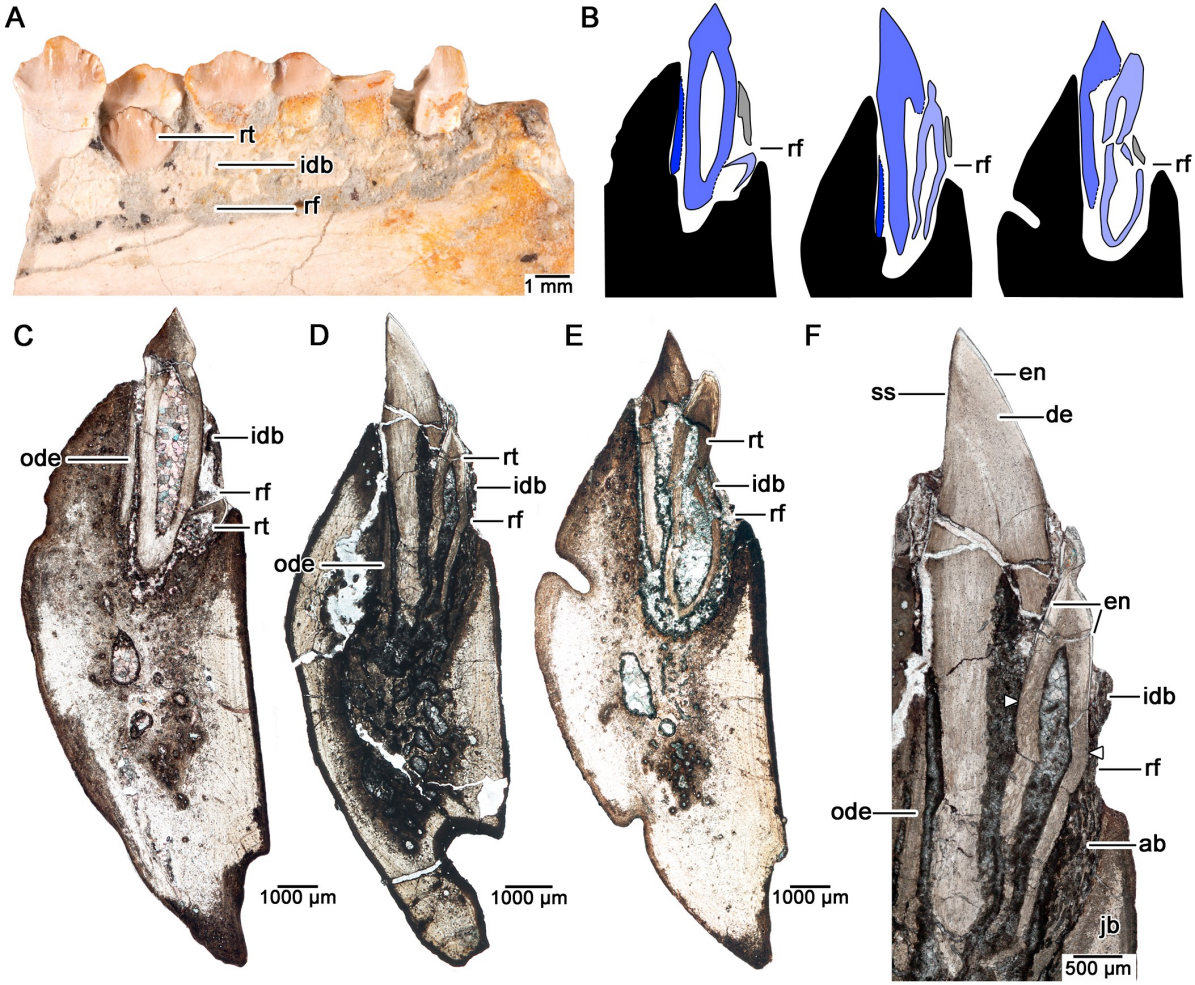
Tooth histology and a reconstruction of the replacement cycle in serial coronal sections of the dentary teeth in *Changchunsaurus parvus*. **A.** Image of the lingual surface of a partial dentary serially sectioned in coronal aspect (JLUM-Ya-C160711D). **B.** A reconstruction of the tooth replacement sequence in *C*. *parvus* based on serial coronal sections through a partial dentary. Sections reveal that the jawbone (black) has a lower lingual wall than the labial wall and this discrepancy is compensated by the interdental bone (grey), which lines lingual surface of the tooth row and the dorsal border of the replacement foramen. Shades of blue indicate the generations of teeth, with darker colours being older generations. Lingual is to the right. **C.** Coronal section (TS01048) of a tooth position showing an early-stage replacement tooth forming adjacent to the replacement foramen (note the root fragment from an older tooth generation along the labial wall of the alveolus). **D.** Coronal section (TS01043) of a later replacement stage where the replacement tooth has resorbed a significant portion of the functional tooth root. The replacement tooth does not fully invade the pulp cavity of its predecessor and instead begins to erupt lingual to the functional tooth. **E.** Coronal section (TS01049) of a slightly later replacement stage where the replacement tooth is nearing eruption lingual to the functional tooth. **F.** Close-up of the teeth in D showing the histological features of the tooth crowns, alveolar bone, and interdental bone. Note the asymmetry in the extent of the enamel cap on the replacement tooth (white arrowheads). **Abbreviations, ab,** alveolar bone; **de,** dentine; **en,** enamel; **idb,** interdental bone; **jb,** bone of the jaw; **ode,** old remnant of dentine; **rf,** replacement foramen; **rt,** replacement tooth; **ss,** shearing surface.

Thin sections through the dentaries of *Chengchunsaurus parvus* revealed teeth at nearly every major stage of tooth development and thus strong evidence for continuous tooth replacement (Figs 1–3). One transverse section across all dentary tooth positions (totaling 15 tooth positions) showed nine tooth positions with either a replacement tooth in the process of resorbing the root of a preceding functional tooth, or the development of a resorption pit adjacent to a functional tooth (Fig 2A). At the earliest stage of the tooth replacement cycle, a new tooth develops lingual to its predecessor at the level of a large foramen that opens onto the lingual surface of the dentary (also termed “special foramina” sensu [38], or “replacement foramina” sensu [42]) (Fig 3), supporting the hypothesis that these foramina are the entry points of a lingually positioned dental lamina into the dentary [38,42]. We prefer the term “replacement foramina” for these features, because of the implied association with the dental lamina. The earliest replacement teeth are adjacent to the replacement foramina and consist of enamel-capped cones of orthodentine (Fig 3A–3C). The crowns of the replacement teeth are labiolingually narrow, but mesiodistally very wide in cross-section (Figs 2C, 3B and 3C). Root resorption begins early in the tooth replacement cycle, because the earliest stages of replacement tooth development are already associated with shallow resorption pits along the roots of the functional teeth (Figs 2C and 3C).

At later stages of tooth replacement, the replacement teeth have complete crowns and elongating roots, but unlike in theropod or hadrosaurid dinosaurs [8], the replacement teeth do not fully invade the pulp chambers of the functional teeth. Instead of breaching the pulp chamber early in development and then erupting vertically as they do in crocodilians and many other dinosaurs [5,8], the replacement teeth spend most of their early development lingual to the functional teeth (Figs 2C, 2D and 3). However, given the relatively large size of the tooth crown compared to the root, the developing tooth still causes extensive root resorption to its functional predecessor (Fig 2A and 2C). By the time a functional tooth is about to be shed, the replacement tooth crown is nearly fully erupted and is visible above the margin of the interdental bone in lingual view (Fig 3A). After the tooth is shed, the erupting tooth must migrate labially to come to the fully functional position. During ontogeny, successive tooth generations may gradually migrate lingually in the dentary of *C*. *parvus*, given that several remnants of previous generations of teeth are embedded in the labial socket wall (Figs 2D and 3B–3F). A similar phenomenon occurs in the dentition of the early neoceratopsian dinosaur *Liaoceratops*, where numerous root fragments are preserved within the jaws due to incomplete root resorption during the replacement cycle, which is a result of gradual tooth migration [24].

### Tooth histology

The enamel in *Chengchunsaurus* teeth is between 30 and 55 μm thick in erupting, unworn replacement teeth (and thus the closest approximation for maximum enamel thickness). In thin section, the enamel cap is the same thickness and roughly symmetrical on the labial and lingual sides of the tooth, unlike the condition in hadrosaurid and ceratopsid dinosaurs [2,6,8,9,13,29]. One thin section revealed that the labial face of the developing tooth crown may have a slightly shorter enamel-covered portion compared to that along the lingual surface, however the difference is subtle (Fig 3F).

**Fig 4:**
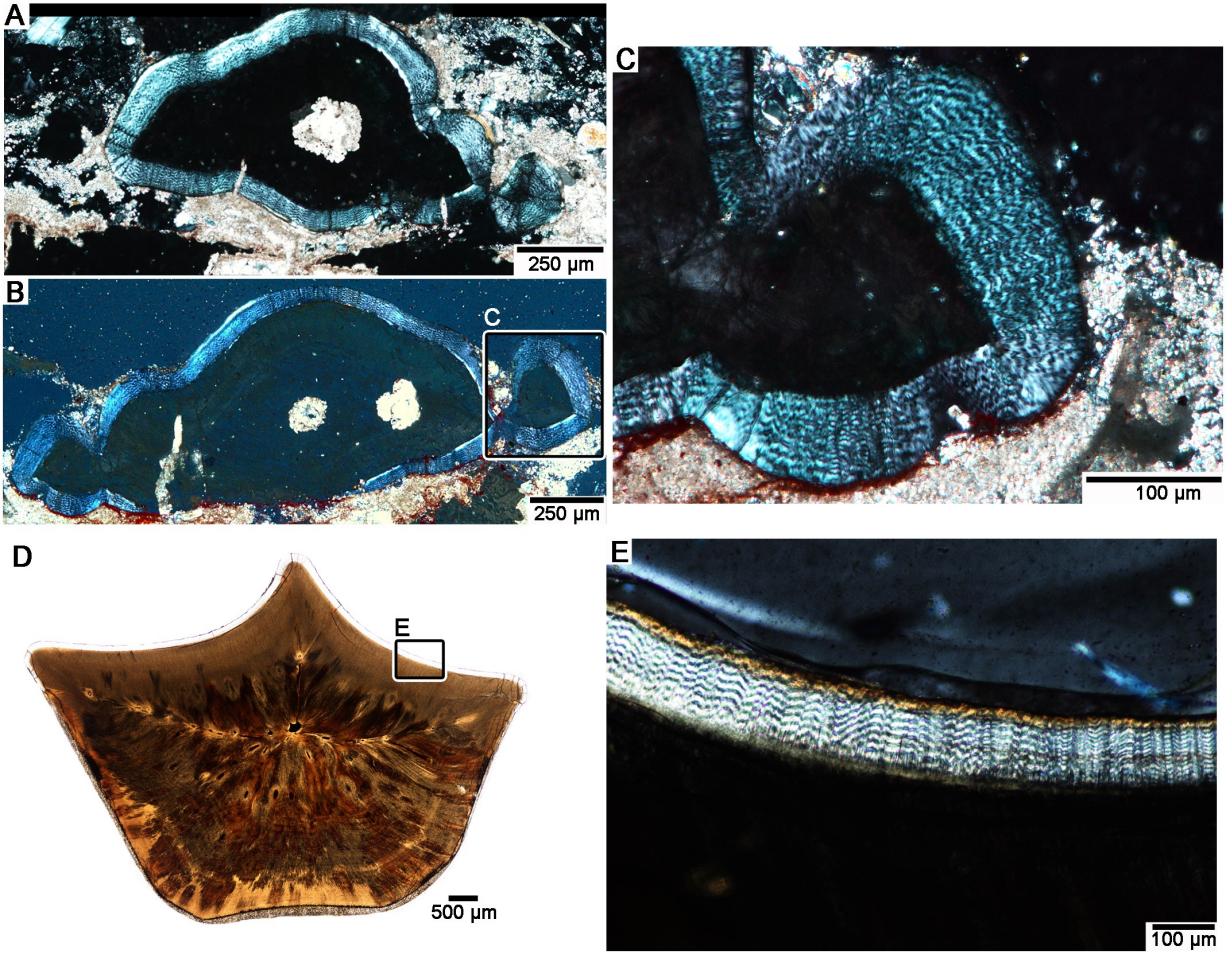
Comparisons of wavy enamel in *Changchunsaurus parvus* and hadrosaurids. **A.** Transverse section through a replacement tooth crown (JLUM Ya-C160711C) under cross-polarized light, showing the wavy enamel in *Changchunsaurus parvus*. **B.** Transverse section (TS01046) through the same tooth in a deeper section. **C.** Close-up of the tooth in B showing the wavy enamel under cross-polarized light. **D.** Transverse section through a hadrosaurid tooth (UALVP 55127). **E.** Close-up of the wavy enamel in a hadrosaurid tooth under cross-polarized light.

**Fig 5:**
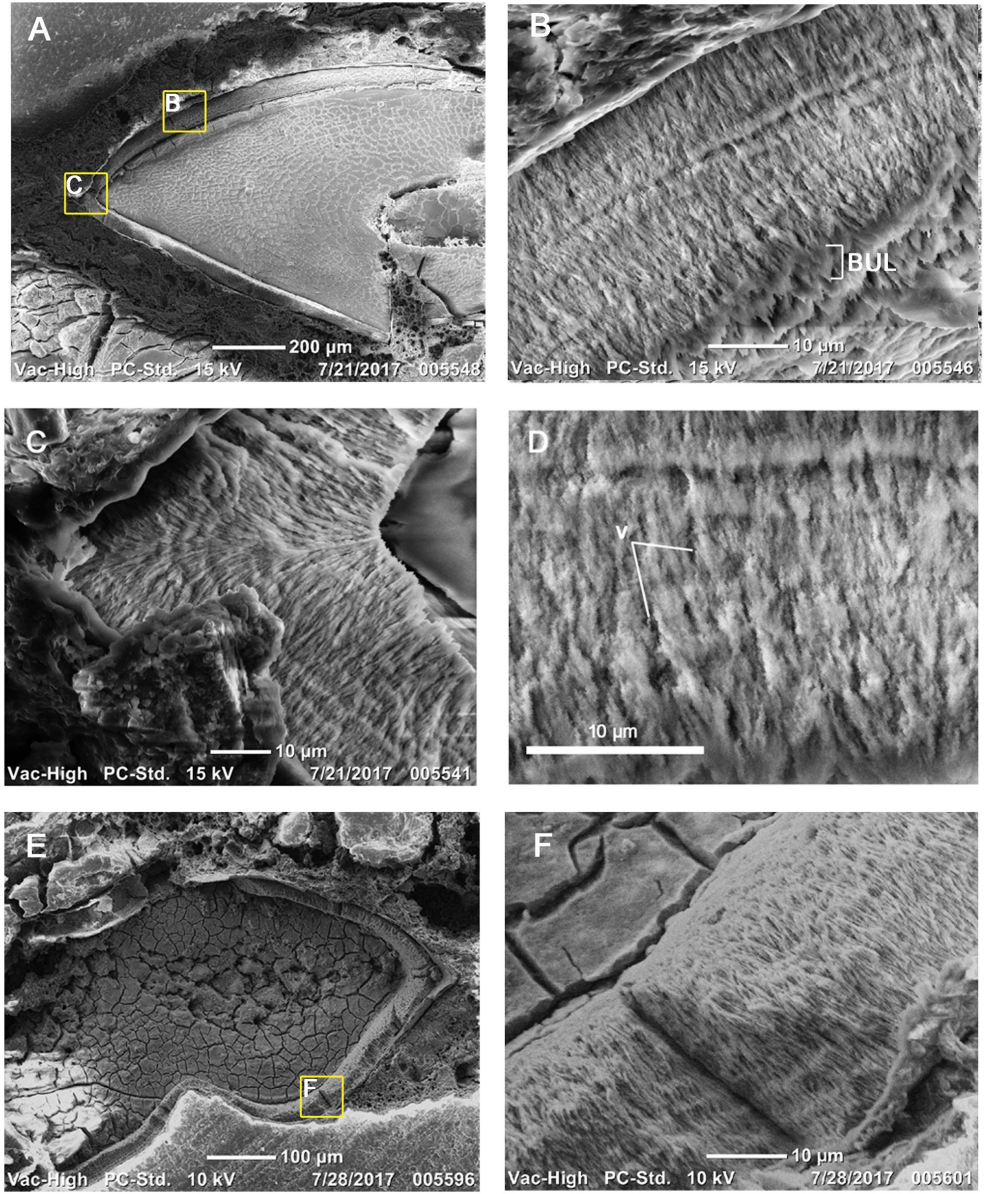
Scanning Electron Microscope images of enamel in replacement teeth of *Changchunsaurus parvus*. **A.** Overview image of the replacement tooth crown (JLUM-Ya-C160711b) in B, C, and D. **B.** Wavy enamel along the lingual surface of a replacement tooth crown in coronal section. **C.** Divergent enamel crystallites on either side of a major discontinuity at the tip of the replacement tooth crown. **D.** Close-up image showing unmineralized voids in the enamel. **E.** Transverse section of a replacement tooth crown (TS01046). **F.** Wavy enamel in transverse section. Note the helical arrangements of the enamel crystallites. **Abbreviations: BUL,** Basal Unit Layer; **EDJ,** Enamel-Dentine Junction; **v,** voids.

Under plane-polarized light, the enamel is clear, but under cross-polarized light, the enamel forms undulating waves that extend parallel to the enamel-dentine junction (EDJ). This pattern is identical to the appearance of hadrosaurid wavy enamel under cross-polarized light (Fig 4). This type of enamel has only been documented elsewhere in dryosauromorph dinosaurs [2,3,22], as well as the rhabdodontid *Matheronodon provincialis* [10]. To confirm the presence of wavy enamel in *Changchunsaurus parvus*, we examined the enamel of replacement tooth crowns under SEM (Fig 5). SEM imaging revealed a relatively straight EDJ and a thin (5–10 μm, up to 18% of total enamel thickness) Basal Unit Layer (BUL) of diverging enamel crystallites at the base of the enamel (Fig 5A). The BUL grades into a much thicker layer (up to 50 μm thick, 91% of enamel thickness) of poorly organized, helically-arranged bundles of enamel crystallites (modules sensu [22]), which are the defining features of wavy enamel [11,22]. The wavy enamel is not as well organized as it is in iguanodontian teeth [3]. Some unmineralized voids are present within the enamel, which are also found in the coarse wavy enamel of iguanodontian dinosaurs [3,22]. Enamel crystallites also diverge from a major discontinuity at the tip of the tooth crown under SEM (Fig 5C).

Very few dentine tubules cross the EDJ into the enamel to form enamel spindles, unlike the condition in hadrosaurids and marginocephalians [12,17]. Fully erupted teeth are quickly incorporated into the shearing surface, presumably immediately after the previous tooth is shed. The enamel on the labial surfaces of the dentary teeth is rapidly worn away due to attrition, exposing a broad plane of orthodentine along the shearing surface (Fig 3F). The orthodentine is similar to that of most other amniotes in that it is avascular, tubular, and is thicker in older teeth due to the centripetal deposition of dentine matrix in life [5,8,43]. The orthodentine along the shearing surfaces of the functional teeth shows no evidence of complex vascular structures or sclerotic dentine, like those proposed for hadrosaurid dinosaurs [2,9]. Instead, the dentine tubules of worn teeth appear open to the surface (Fig 3F). The dentine also contains numerous concentric growth lines, which are spaced approximately 22–17 μm apart. The spacing between successive lines is consistent with daily lines of von Ebner in other archosaurs [1,44]. Unfortunately, the dentine is too diagenetically modified to examine the prevalence of von Ebner lines in functional and replacement teeth at a single tooth position to gauge the tooth replacement rate for this taxon.

**Fig 6:**
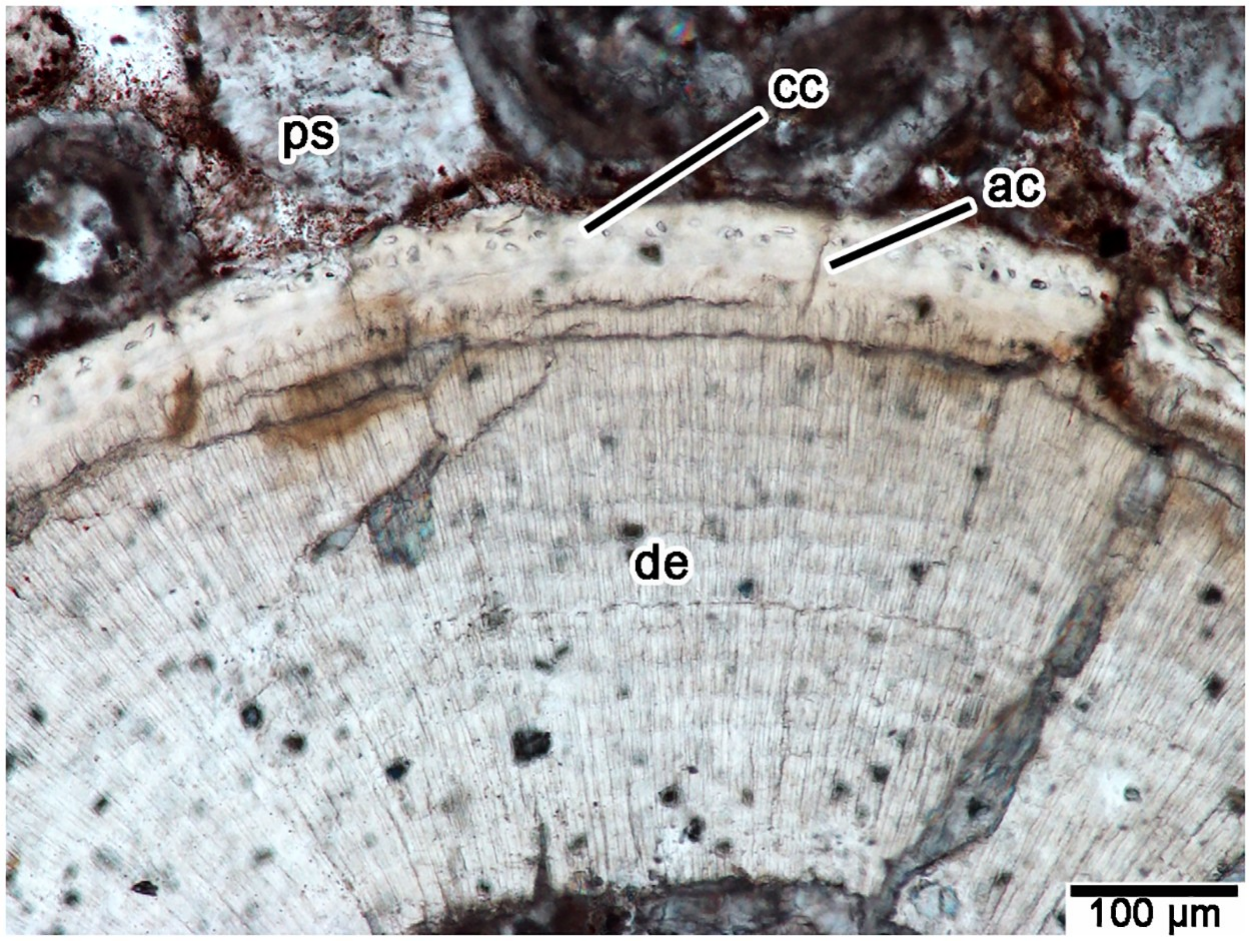
Tooth attachment tissues in transverse section (TS01032) of a *Changchunsaurus parvus* tooth root (JLUM Ya-C160711e2). **Abbreviations: ac**, acellular cementum; **cc**, cellular cementum; **de**, dentine; **ps**, periodontal space.

The teeth of *Changchunsaurus parvus*, like those of all other dinosaurs where tooth attachment tissues are described [5,7,8,23], were attached to the sockets by a periodontal ligament (a gomphosis-type attachment). In section, every tooth root is separated from the surrounding socket bone by a mineral-filled space. In modern crocodilians, this space houses the periodontal ligament in life [6,45–47]: a network of collagen fiber bundles that anchor into the walls of the tooth socket and the surface of the tooth root. The surfaces of the tooth roots in *Changchunsaurus parvus* are coated in a thin band of acellular cementum, and a thicker, outer layer of cellular cementum (Fig 6), which would have been the anchoring points for the periodontal ligament [8]. The outer layer of cellular cementum is relatively thin compared to that of hadrosaurid and ceratopsid dinosaurs and is avascular, which is more similar to the condition in theropods [5,8,23]. Alveolar bone forms the tooth socket in all amniotes [8,14,48–50] and is composed of highly vascular bone, which is separated from the surrounding bone of the jaw by a reversal line (Figs 2 and 3F). Unfortunately, the alveolar bone is heavily diagenetically modified and the exact bone matrix type was indiscernible under cross-polarized light. Given its similarity to the microcancellous alveolar bone of other dinosaurs, however, it is likely that this tissue was a rapidly deposited type of woven bone [8]. In transverse section, the alveolar bone encircles each tooth, but is most extensive in between tooth positions and along the labial margins (Fig 2). In coronal section, the interdental bone forming the margin of the lingual wall of the socket is made entirely of alveolar bone, and not jawbone, similar to the interdental plates and bone in other dinosaurs [8].

The bone of the dentary is poorly vascularized. In transverse section, the dentary bone forms a thick labial wall and a thin lingual wall (in sections taken below the interdental bone) (Fig 2). In coronal section, the dentary bone is asymmetrical around the tooth; the labial wall reaches a level just below the shearing surfaces of the teeth, whereas the lingual wall is much shorter, extending only to the ventral margins of the replacement foramina (Fig 3).

## Discussion

### Insights into dental evolution in ornithopod dinosaurs

Dental innovations have been a focal point in ornithischian research, particularly because of the evolution of complex dental batteries in hadrosaurid and ceratopsid dinosaurs [2,6,9,13,31,51]. Hadrosaurid teeth in particular are touted as the most histologically complex of reptilian teeth [2], because of the presence of novel dental tissues (e.g. coronal cementum, longitudinally and transversely oriented “giant tubules”). This view was challenged more recently [6,8,9], because dinosaur dental tissue homology, development, and evolution have only begun to be studied in other groups and compared to hadrosaurids. What remains is a need for histological analyses of early members of ornithischian clades in order to reconstruct the evolutionary origins of such complex structures.

Regardless of its purported phylogenetic position as an early ornithopod [40] or as an earlier ornithischian [41], *Changchunsaurus parvus* is thus far our best approximation of the ancestral condition for ornithopod dental development and histology and therefore has important bearing on our understanding of dental evolution in the group. In most respects, the histological properties of the teeth of *Changchunsaurus* are similar to those of other dinosaurs. Their teeth, like those of theropods [5,8,23], sauropods [7], and other ornithischians [6,9,13] are coated in layers of cementum, which provide the anchoring points for the periodontal ligament (PDL). This ligament suspends each tooth within its socket, providing mobility and flexibility at the tooth-socket interface. As is typical of ornithischian dinosaurs [9,35], the pulp chamber is not exposed along the shearing surface, although there are no specializations for rapid pulp closure as there are in iguanodontians [2,9,35].

**Fig 7:**
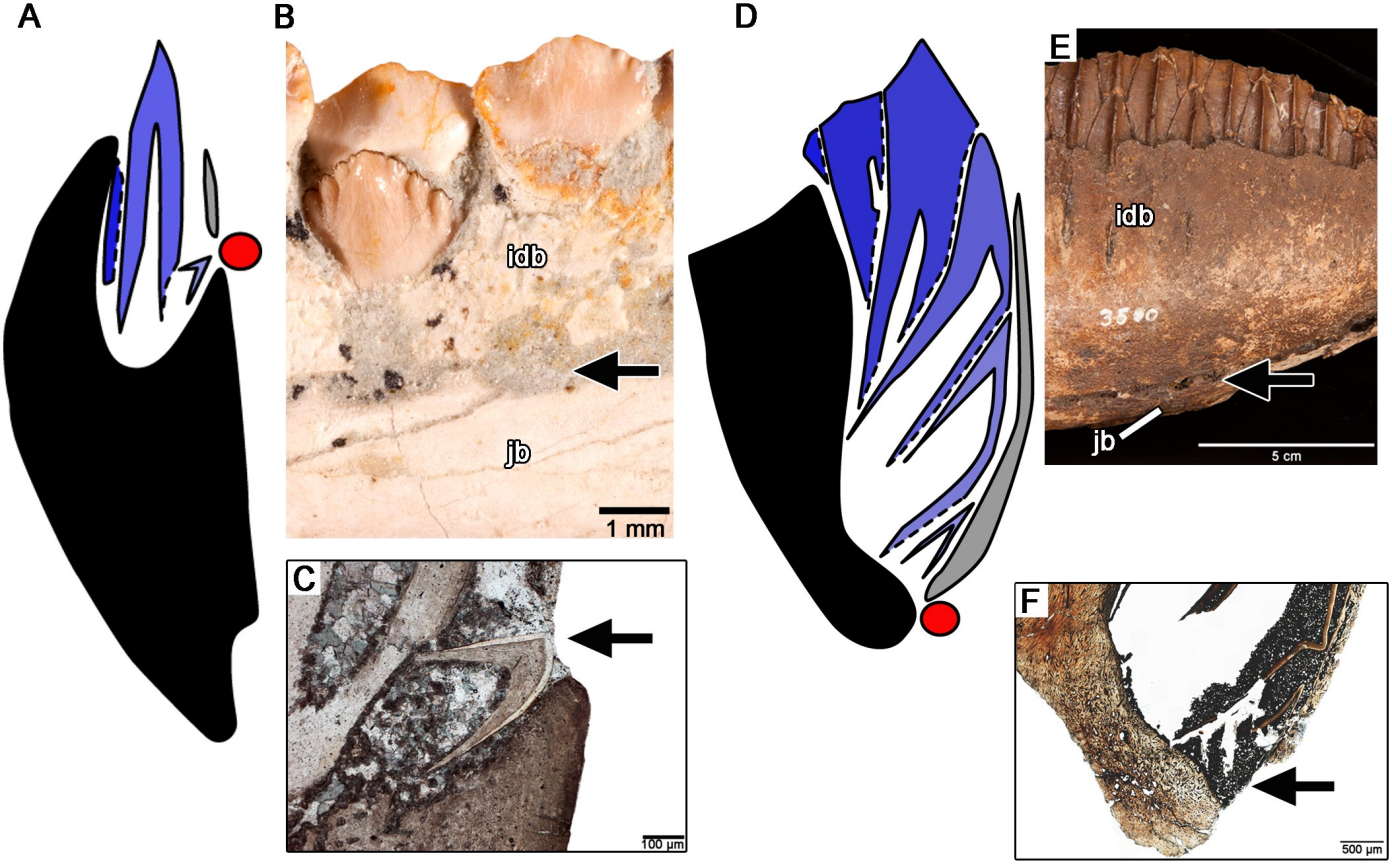
Comparisons of tooth development and jaw geometry in *Changchunsaurus parvus* and a hadrosaurid. **A.** Tooth implantation and replacement in *C*. *parvus*. Bone of the jaw is indicated in black, successive tooth generations in blue, position of dental lamina in red and interdental bone in grey. **B.** Closeup of the lingual surface of the dentary in *C*. *parvus* (JLUM Ya-C160711d) showing the positions of the interdental bone and replacement foramina. **C.** Closeup of a developing tooth at the opening of the replacement foramen in thin section (TS0 1048) in *C*. *parvus*. **D.** Tooth implantation and replacement in a hadrosaurid dentary. Bone of the jaw is indicated in black, successive tooth generations in blue, position of dental lamina in red and interdental bone in grey. **E.** Lingual view of a hadrosaurid dentary (ROM 3500) showing position of the interdental bone and replacement foramina. **F.** Closeup of the replacement foramen at the base of a dental battery (ROM 3500) in thin section. Black arrows indicate positions of replacement foramina. **Abbreviations: idb,** interdental bone; **jb,** bone of the jaw.

Our thin sections of *C*. *parvus* also support the function of the replacement foramina along the lingual surfaces of the jaws as the entry points for an externally placed dental lamina, a condition previously hypothesized for the similarly placed “special foramina” in other ornithischians [9,38]. Unlike the condition in modern crocodilians, in which the dental lamina is buried within the jaws, the earliest forming replacement teeth in *C*. *parvus* are found adjacent to replacement foramina that open to the lingual surface of the dentary. The ventral margins of these foramina are lined by the bone of the jaw, whereas the remaining border is formed by short extensions of interdental bone (Figs 3 and 7). In coronal sections, the bone of the jaw thus forms a slightly asymmetrical support around the teeth and the teeth and alveolar tissues are restricted to the dorsal half of the dentary (Fig 7).

In hadrosaurids, the replacement foramina are situated farther away from the occlusal margin than in *C*. *parvus*, but they are still associated with the newest generations of replacement teeth [8,38]. Given that these foramina likely mark the position of the dental lamina, we hypothesize that one major difference between early ornithopod and hadrosaurid dental development was the change in position of the dental lamina from close to the apex of the jaw in early ornithopods to the base of the lingual surface of the tooth-bearing element in hadrosaurids (Fig 7). This heterotopic shift in the site of tooth initiation had two consequences in hadrosaurid dental evolution. The first is the nearly complete reduction of the lingual wall of the jawbones (the entire lingual wall of the jaws in hadrosaurids is composed of interdental bone, and not jawbone [8,9]). The second is that the teeth and alveolar tissues extend through the entire depth of the dentary and maxilla (Fig 7) [8,9]. It is worth noting, however, that replacement foramina are variably present in other ornithischian dinosaurs [34,35,41], suggesting that there may be variation in the location of the dental lamina in many groups.

Herbivorous dinosaurs also faced the challenge of extensive tooth wear due to their abrasive diets [2,13]. Sauropods, neoceratopsians, and hadrosaurids circumvented this issue by maintaining a constantly replenishing grinding or shearing surface via rapid and coordinated tooth replacement [1,24–27,52,53]. Interestingly, *Changchunsaurus parvus* shows evidence of continuous tooth replacement, despite maintaining a fairly uniform shearing surface of imbricated tooth crowns to pulverize tough plant material [42]. Continuous tooth replacement in these types of dentitions should typically leave gaps along the shearing surface, which would be detrimental for an herbivorous dinosaur. However, as noted by previous authors (e.g. [35]) such gaps are typically absent. This may be the result of a unique replacement mode, which is typified by that in *C*. *parvus* (Figs 2 and 3). Unlike in modern crocodilians or in theropod dinosaurs [5,8], a developing replacement tooth in *C*. *parvus* never fully invades the pulp chamber of the preceding functional tooth until it has almost completely erupted (Figs 2 and 3). This would be advantageous for a shearing dentition, because it would delay the shedding of the older functional tooth until its replacement is nearly complete and in proper position. Whether this replacement mode is unique to *Changchunsaurus* among ornithischians is unclear, but merits future study to see if it evolved in association with a shearing dentition.

Finally, our report of wavy enamel in *Changchunsaurus parvus* is the phylogenetically earliest occurrence of this enamel type, however it is not the oldest chronological record of this tissue. Wavy enamel has also been found in the iguanodont *Dryosaurus altus* from the Late Jurassic of the United States [3]. The microstructure of wavy enamel was first described by Sander [22] in *Iguanodon* and the hadrosaurid *Anatosaurus*. Combined with the absence of wavy enamel in other sampled ornithischians, Sander [22] concluded that this peculiar enamel type evolved in association with more complex dental occlusion and secondary wear surfaces in iguanodontians. Subsequently, Hwang [3] identified wavy enamel in *Dryosaurus*, *Camptosaurus*, *Prosaurolophus*, and *Saurolophus* and noted differences in the wavy enamel subtypes in progressively more derived iguanodontians. Hwang [3] hypothesized that wavy enamel arose and subsequently co-evolved with a suite of dental characters in early iguanodontians, culminating in the dental batteries of hadrosaurids.

**Fig 8:**
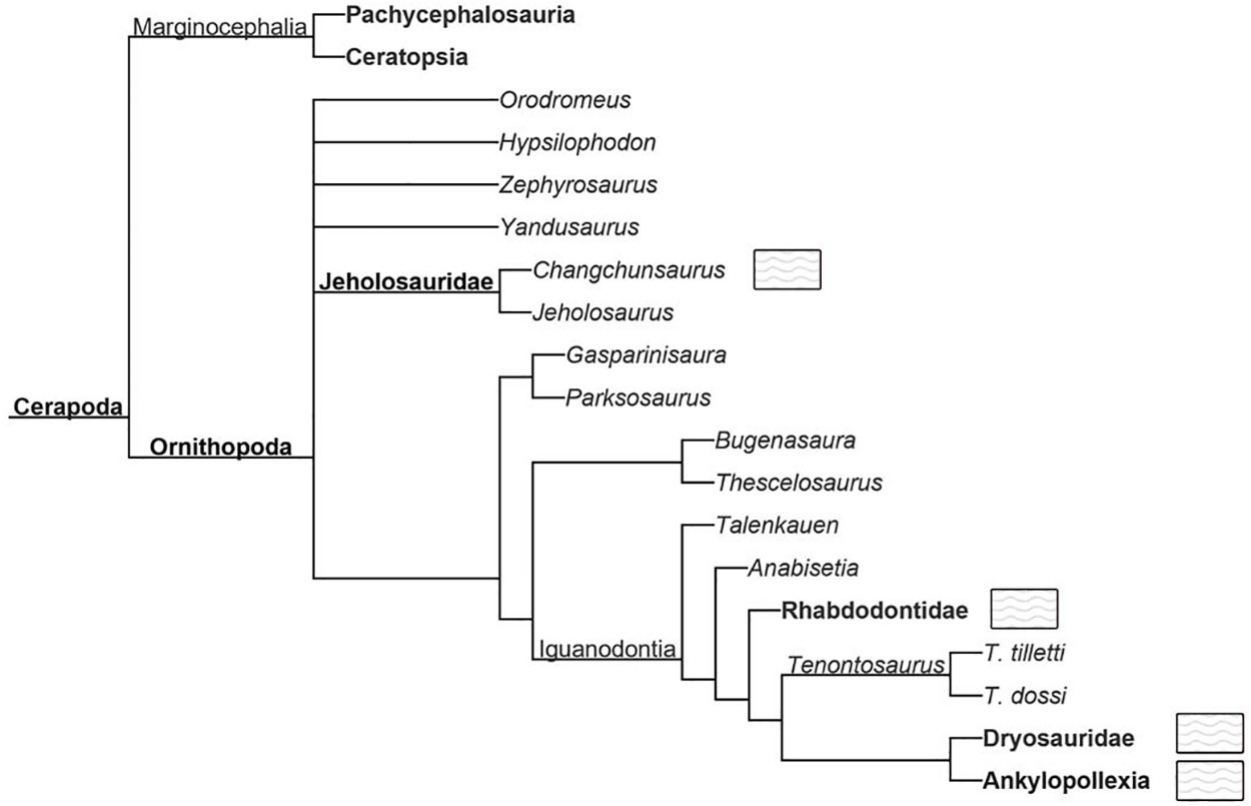
Distribution of wavy enamel in Ornithopoda (phylogeny modified from [40]). Boxed symbols indicate presence of wavy enamel in *Changchunsaurus* (this study), Rhabdodontidae [10], Dryosauridae [3], Ankylopollexia [3,4,22].

The presence of wavy enamel in the shearing teeth of the rhabdodontid *Matheronodon provincialis* [10] and now the discovery of wavy enamel in *Changchunsaurus parvus* casts doubt on previous evolutionary hypotheses regarding the origin of this unique reptilian enamel type (Fig 8). The teeth of *C*. *parvus*, like those of ‘heterodontosaurids’ and early ornithopods more generally, have expanded, denticulated, and ornamented tooth crowns that are organized into a single tooth row in each jaw quadrant [34,35,42,54]. Moreover, the shearing surfaces of the teeth are simple, flat or gently concave surfaces supported by avascular orthodentine, bearing no resemblance to the complex occlusal plane morphology in hadrosaurid dinosaurs (Fig 3) [2]. What is surprising is that these large, leaf-shaped teeth already possess an enamel type that was previously considered as an iguanodontian (or dryomorphan) synapomorphy [3,22].

So far, we can only conclude that this wavy enamel type evolved in association with a shearing-type dentition with a roughly symmetrically-enamelled crown, although its precise function is still unclear. In *Changchunsaurus*, the “waves” of enamel are present on all sides of the tooth crowns, including the surface that is worn away during feeding (Figs 1, 4 and 5). In this region of the crown, the “waves” are oriented roughly parallel to the plane of tooth wear and the enamel is eventually worn away. At the apex of a worn tooth crown, the enamel “waves” are obliquely oriented relative to the wear surface and may impart more wear resistance, thus forming a small crest of enamel (Fig 1).

### A future role for dental histology in reconstructing ornithischian phylogeny

Studying the histological features of the teeth of *Changchunsaurus parvus* presents a unique opportunity to address broader questions in dinosaurian phylogeny and the role of histology in future character construction. *C*. *parvus* was originally classified as ‘Ornithopoda *incertae sedis*’, because this taxon possesses a number of features consistent with early ornithopods, marginocephalians, and even cerapodans more broadly [39,42]. Butler et al. [54] noted that basal cerapodan interrelationships represented one of the most problematic and poorly understood areas of study in ornithischian phylogeny, further highlighting the importance of phylogenetically early forms, including *C*. *parvus*. Butler et al. [40] subsequently provided the first phylogenetic hypothesis for the placement of *C*. *parvus* within Ornithopoda, forming a sister group relationship with the contemporaneous *Jeholosaurus shangyuanensis*, a clade that was in turn sister to all other members of Ornithopoda. Butler et al. [40], however, noted that the relationships of early cerapodans remain poorly supported and thus their placement within Ornithopoda was still provisional. By comparison, Boyd [41] placed *Changchunsaurus* within a new family (Parksosauridae), which was the sister taxon to all Cerapoda.

The importance of phylogenetic relationships of early members of the major dinosaurian clades is now more important than ever to the broader understanding of dinosaur evolution [34,41,55]. The need for phylogenetically informative characters is thus paramount to this issue, and, given the significance of the feeding apparatus to the success of ornithischian clades [28,56,57], tooth characters often figure prominently in phylogenetic analyses. For example, the character list used by Butler et al. [54] consisted of 221 characters, 21 (9.5%) of which concerned the size, number, ornamentation, or replacement of teeth. 30 of the 255 characters (11.7%) used in Boyd’s [41] analysis of Ornithischia are dental characters. Histology could provide additional support for, or refinements to tooth characters, but also yield new information for character construction.

We highlight the need to incorporate enamel microstructure into phylogenetic analyses of Ornithischia. Wavy enamel was previously hypothesized to be a synapomorphy of iguanodontians [3], but the discovery of this enamel type in *Changchunsaurus* pushes this feature back to a potentially important evolutionary split between ornithopods and marginocephalians (Fig 8). This depends on future study of enamel microstructure in early marginocephalians and euornithopods, given the reported absence of wavy enamel in ceratopsians, pachycephalosaurids, *Thescelosaurus* and *Tenontosaurus* [3].

Given the contentious placement of *Changchunsaurus* within ornithischian phylogeny [40,41], more detailed taxonomic sampling is required to determine if wavy enamel evolved independently in *C*. *parvus* or if it indeed evolved much earlier than previously thought and is a symplesiomorphy in Iguanodontia. In our opinion, future studies need to search for the presence of wavy enamel first using thin sections and cross-polarized light microscopy, given the ease with which wavy enamel can be identified using this technique (Figs 1 and 4) [2,10]. The wavy enamel subtypes (sensu [3]) can then be determined using SEM images of acid-etched surfaces. The reason for this two-step approach is because the wavy enamel found in *C*. *parvus*, similar to that of *Dryosaurus*, is actually difficult to differentiate from columnar enamel under SEM, because it is poorly organized and the helical arrangements of the enamel crystallites are more subtle [3]. Thus, the presence of wavy enamel is better assessed at lower magnifications and under cross-polarized light, followed by more detailed investigation of wavy enamel subtypes (“rudimentary”, coarse, fine, sensu [3,22]) under SEM.

Other dental features that may be useful in phylogenetic analyses of ornithischians include: the relative contribution of interdental bone to the lingual surface of the jaw, the presence of vascularized cellular cementum along the tooth roots (vascular in hadrosaurids and ceratopsids examined thus far, and avascular in other dinosaurs), the tooth replacement mode [8], and adaptations for rapid dental pulp closure [2,9,35]. In many cases, these features can be evaluated non-destructively with high-resolution CT or synchrotron scans (e.g. [23,34]), but wherever needed, thin section data could be used to validate these observations.

## Conclusions

Given the previous focus on dental histology in derived, Late Cretaceous carnivores and herbivores in the literature [5], the present histological analysis of the teeth and jaws of *Changchunsaurus parvus* from the Cretaceous Quantou formation in Jilin Province, China, provides the first in-depth look at tooth histology and development in a dinosaur that may lie close to the base of the ornithopod radiation [40]. This has important bearing on our understanding of the evolution of the more complex dental batteries seen in hadrosaurids from the more modest, single-rowed dentitions of most herbivorous dinosaurs. These histological sections have revealed several surprising features that may have evolved in association with herbivory in this early ornithischian, including the presence of wavy enamel and an unusual tooth replacement mode. In contrast, the presence of root cementum and a periodontal ligament connection of the tooth to the alveolus are probably symplesiomorphic features in ornithischians and in dinosaurs more generally. The presence of replacement foramina close to the apex of the jaw and a short contribution of interdental bone contrasts strongly with the condition in hadrosaurids. We use this comparison to hypothesize that hadrosaurids have evolved a reduced lingual wall of the jaws and, correspondingly, greater contributions of interdental bone via a shift in the position of the dental lamina towards the base of the jaws. Using these data as an example, we advocate that similar studies of early members of major dinosaurian clades are needed in order to incorporate histological characters into phylogenetic analyses and to understand how dinosaur dentitions have evolved over millions of years to cope with their diverse diets.

## References

[1] Erickson GM. Incremental lines of von Ebner in dinosaurs and the assessment of tooth replacement rates using growth line counts. Proc Natl Acad Sci. 1996;93: 14623–14627. 8962103 10.1073/pnas.93.25.14623 PMC26184

[2] Erickson GM, Krick BA, Hamilton M, Bourne GR, Norell MA, Lilleodden E, et al Complex Dental Structure and Wear Biomechanics in Hadrosaurid Dinosaurs. Science. 2012;338: 98–101. 10.1126/science.1224495 23042891

[3] Hwang SH. The evolution of dinosaur tooth enamel microstructure. Biol Rev. 2011;86: 183–216. 10.1111/j.1469-185X.2010.00142.x 20518758

[4] Hwang SH. Phylogenetic patterns of enamel microstructure in dinosaur teeth. J Morphol. 2005;266: 208–240. 10.1002/jmor.10372 16163689

[5] Fong RKM, LeBlanc ARH, Berman DS, Reisz RR. Dental histology of *Coelophysis bauri* and the evolution of tooth attachment tissues in early dinosaurs: Dinosaur Dental Histology. J Morphol. 2016;277: 916–924. 10.1002/jmor.20545 27087142

[6] Bramble K, LeBlanc ARH, Lamoureux DO, Wosik M, Currie PJ. Histological evidence for a dynamic dental battery in hadrosaurid dinosaurs. Sci Rep. 2017;7 10.1038/s41598-017-16056-3 29150664 PMC5693932

[7] García RA, Zurriaguz V. Histology of teeth and tooth attachment in titanosaurs (Dinosauria; Sauropoda). Cretac Res. 2016;57: 248–256. 10.1016/j.cretres.2015.09.006

[8] LeBlanc ARH, Brink KS, Cullen TM, Reisz RR. Evolutionary implications of tooth attachment versus tooth implantation: A case study using dinosaur, crocodilian, and mammal teeth. J Vertebr Paleontol. 2017; e1354006 10.1080/02724634.2017.1354006

[9] LeBlanc ARH, Reisz RR, Evans DC, Bailleul AM. Ontogeny reveals function and evolution of the hadrosaurid dinosaur dental battery. BMC Evol Biol. 2016;16 10.1186/s12862-016-0721-1 27465802 PMC4964017

[10] Godefroit P, Garcia G, Gomez B, Stein K, Cincotta A, Lefèvre U, et al Extreme tooth enlargement in a new Late Cretaceous rhabdodontid dinosaur from Southern France. Sci Rep. 2017;7 10.1038/s41598-017-13160-2 29074952 PMC5658417

[11] Hwang SH. The utility of tooth enamel microstructure in identifying isolated dinosaur teeth. Lethaia. 2009;43: 307–322. 10.1111/j.1502-3931.2009.00194.x

[12] Wang C-C, Song Y-F, Song S-R, Ji Q, Chiang C-C, Meng Q, et al Evolution and Function of Dinosaur Teeth at Ultramicrostructural Level Revealed Using Synchrotron Transmission X-ray Microscopy. Sci Rep. 2015;5 10.1038/srep15202 26512629 PMC4625602

[13] Erickson GM, Sidebottom MA, Kay DI, Turner KT, Ip N, Norell MA, et al Wear biomechanics in the slicing dentition of the giant horned dinosaur *Triceratops*. Sci Adv. 2015;1: e1500055–e1500055. 10.1126/sciadv.1500055 26601198 PMC4640618

[14] Reid REH. Bone histology of the Cleveland-Lloyd dinosaurs and of dinosaurs in general, Part I: Introduction: Introduction to bone tissues. Brigh Young Univ Geol Stud. 1996;41: 25–72.

[15] Erickson GM, Zelenitsky DK, Kay DI, Norell MA. Dinosaur incubation periods directly determined from growth-line counts in embryonic teeth show reptilian-grade development. Proc Natl Acad Sci. 2017;114: 540–545. 10.1073/pnas.1613716114 28049837 PMC5255600

[16] Erickson GM, Zelenitsky DK. Osteohistology and occlusal morphology of *Hypacrosaurus stebengeri* teeth throughout ontogeny with comments on wear-induced form and function In: Eberth DA, Evans DC, editors. Hadrosaurs. Bloomington, Indiana, U. S. A: Indiana University Press; 2014 pp. 422–432.

[17] Brink KS, Reisz RR, LeBlanc ARH, Chang RS, Lee YC, Chiang CC, et al Developmental and evolutionary novelty in the serrated teeth of theropod dinosaurs. Sci Rep. 2015;5: 12338 10.1038/srep12338 26216577 PMC4648475

[18] Abler WL. The serrated teeth of tyrannosaurid dinosaurs, and biting structures in other animals. Paleobiology. 1992;18: 161–183.

[19] Buffetaut E, Dauphin Y, Jaeger JE, Martin M, Mazin J-M, Tong H. Prismatic enamel in theropod dinosaurs. Naturwissenschaften. 1986;73: 326–327. 3748191 10.1007/BF00451481

[20] Zanno LE, Makovicky PJ. On the earliest record of Cretaceous tyrannosauroids in western North America: implications for an Early Cretaceous Laurasian interchange. Hist Biol. 2011;23: 317–325.

[21] Stokosa K. Enamel microstructure variation within the Theropoda In: Carpenter KH, editor. The Carnivorous Dinosaurs. Bloomington, Indiana, U. S. A: Indiana University Press; 2005 pp. 163–178.

[22] Sander PM. The microstructure of reptilian tooth enamel: terminology, function, and phylogeny. Münch Geowiss Abh. 1999;38: 1–102.

[23] Dumont M, Tafforeau P, Bertin T, Bhullar B-A, Field D, Schulp A, et al Synchrotron imaging of dentition provides insights into the biology of *Hesperornis* and *Ichthyornis*, the “last” toothed birds. BMC Evol Biol. 2016;16 10.1186/s12862-016-0753-6 27659919 PMC5034473

[24] He Y, Makovicky PJ, Xu X, You H. High-resolution computed tomographic analysis of tooth replacement pattern of the basal neoceratopsian *Liaoceratops yanzigouensis* informs ceratopsian dental evolution. Sci Rep. 2018;8 10.1038/s41598-018-24283-5 29651146 PMC5897341

[25] Sereno PC, Wilson JA, Witmer LM, Whitlock JA, Maga A, Ide O, et al Structural Extremes in a Cretaceous Dinosaur. PLoS ONE. 2007;2: e1230 10.1371/journal.pone.0001230 18030355 PMC2077925

[26] Schwarz D, Kosch JCD, Fritsch G, Hildebrandt T. Dentition and tooth replacement of *Dicraeosaurus hansemanni* (Dinosauria, Sauropoda, Diplodocoidea) from the Tendaguru Formation of Tanzania. J Vertebr Paleontol. 2015; e1008134 10.1080/02724634.2015.1008134

[27] Edmund AG. Tooth replacement phenomena in the lower vertebrates. R Ont Mus Life Sci Div Contrib. 1960;52: 1–190.

[28] Norman DB. Basal Iguanodontia In: Weishampel DB, Dodson P, Osmólska H, editors. The Dinosauria. Second Berkeley, California: University of California Press; 2004 pp. 413–437.

[29] Horner JR, Weishampel DB, Forster CA. Hadrosauridae In: Weishampel DB, Dodson P, Osmólska H, editors. The Dinosauria. Second Berkeley, California: University of California Press; 2004 pp. 325–334.

[30] Ostrom JH. Functional morphology and evolution of the ceratopsian dinosaurs. Evolution. 1966;20: 290–308. 10.1111/j.1558-5646.1966.tb03367.x 28562975

[31] Strickson E, Prieto-Márquez A, Benton MJ, Stubbs TL. Dynamics of dental evolution in ornithopod dinosaurs. Sci Rep. 2016;6 10.1038/srep28904 27412496 PMC4944125

[32] Norman DB, Witmer LM, Weishampel DB. Basal Ornithischia The Dinosauria. Second Berkeley, California: University of California Press; 2004 pp. 325–334.

[33] Owen R. Odontography; or, a Treatise on the Comparative Anatomy of the Teeth; Their Physiological Relations, Mode of Development, and Microscopic Structure, in the Vertebrate Animals. London: Hippolyte Bailliere; 1840 PMC5592592 30161728

[34] Butler RJ, Porro LB, Galton PM, Chiappe LM. Anatomy and Cranial Functional Morphology of the Small-Bodied Dinosaur *Fruitadens haagarorum* from the Upper Jurassic of the USA. Farke AA, editor. PLoS ONE. 2012;7: e31556 10.1371/journal.pone.0031556 22509242 PMC3324477

[35] Sereno PC. Taxonomy, morphology, masticatory function and phylogeny of heterodontosaurid dinosaurs. ZooKeys. 2012;226: 1–225. 10.3897/zookeys.223.2840 PMC3491919 23166462

[36] Thulborn RA. Tooth wear and jaw action in the Triassic ornithischian dinosaur *Fabrosaurus*. J Zool. 1971;164: 165–179.

[37] Thulborn RA. Aestivation among ornithopod dinosaurs of the African Trias. Lethaia. 1978;11: 185–198.

[38] Edmund AG. On the foramina in the jaws of many ornithischian dinosaurs. Contrib R Ont Mus Div Zool Palaeontol. 1957;48: 1–14.

[39] Zan S-Q, Jun C, Jin L-Y, Li T. A primitive ornithopod from the Early Cretaceous Quantou Formation of central Jilin, China. Vertebr Palasiat. 2005;43: 182–193.

[40] Butler RJ, Liyong J, Jun C, Godefroit P. The postcranial osteology and phylogenetic position of the small ornithischian dinosaur *Changchunsaurus parvus* from the Quantou Formation (Cretaceous: Aptian–Cenomanian) of Jilin Province, North-Eastern China. Palaeontology. 2011;54: 667–683.

[41] Boyd CA. The systematic relationships and biogeographic history of ornithischian dinosaurs. PeerJ. 2015; 1523: 1–62. 10.7717/peerj.1523 PMC4690359 26713260

[42] Liyong J, Jun C, Shu-Qin Z, Butler RJ, Godefroit P. Cranial anatomy of the small ornithischian dinosaur *Changchunsaurus parvus* from the Quantou Formation (Cretaceous: APtian–Cenomanian) of Jilin Province, northeastern China. J Vertebr Paleontol. 2010;30: 196–214.

[43] Berkovitz B, Shellis P. The Teeth of Non-Mammalian Vertebrates. London, United Kingdom: Academic Press; 2017.

[44] Erickson GM. Daily deposition of dentine in juvenile *Alligator* and assessment of tooth replacement rates using incremental line counts. J Morphol. 1996;228: 189–194. 10.1002/(SICI)1097-4687(199605)228:2<189::AID-JMOR7>3.0.CO;2-0 29852586

[45] McIntosh JE, Anderton X, Flores-De-Jacoby L, Carlson DS, Shuler CF, Diekwisch TGH. Caiman periodontium as an intermediate between basal vertebrate ankylosis-type attachment and mammalian"true" periodontium. Microsc Res Tech. 2002;59: 449–459. 10.1002/jemt.10222 12430171

[46] Kvam T. The teeth of *Alligator mississippiensis* (Daud) VI. Periodontium. Acta Odontol Scand. 1960;18: 67–82. 10.3109/00016356209026115 13927728

[47] LeBlanc ARH, Lamoureux DO, Caldwell MW. Mosasaurs and snakes have a periodontal ligament: timing and extent of calcification, not tissue complexity, determines tooth attachment mode in reptiles. J Anat. 2017; 10.1111/joa.12686 28901023 PMC5696141

[48] Caldwell MW. Ontogeny, anatomy and attachment of the dentition in mosasaurs (Mosasauridae: Squamata). Zool J Linn Soc. 2007;149: 687–700.

[49] LeBlanc ARH, Reisz RR. Periodontal Ligament, Cementum, and Alveolar Bone in the Oldest Herbivorous Tetrapods, and Their Evolutionary Significance. Viriot L, editor. PLoS ONE. 2013;8: e74697 10.1371/journal.pone.0074697 24023957 PMC3762739

[50] Ten Cate AR, Mills C. The development of the periodontium: the origin of alveolar bone. Anat Rec. 1972;173: 69–77. 10.1002/ar.1091730106 5028065

[51] Suarez CA, You H-L, Suarez MB, Li D-Q, Trieschmann JB. Stable Isotopes Reveal Rapid Enamel Elongation (Amelogenesis) Rates for the Early Cretaceous Iguanodontian Dinosaur *Lanzhousaurus magnidens*. Sci Rep. 2017;7 10.1038/s41598-017-15653-6 29127359 PMC5681512

[52] Ostrom JH. Cranial morphology of the hadrosaurian dinosaurs of North America. Bull Am Mus Nat Hist. 1961;122: 1–186.

[53] D’Emic MD, Whitlock JA, Smith KM, Fisher DC, Wilson JA. Evolution of High Tooth Replacement Rates in Sauropod Dinosaurs. Evans AR, editor. PLoS ONE. 2013;8: e69235 10.1371/journal.pone.0069235 23874921 PMC3714237

[54] Butler RJ, Upchurch P, Norman DB. The phylogeny of the ornithischian dinosaurs. J Syst Palaeontol. 2008;6: 1–40.

[55] Baron MG, Norman DB, Barrett PM. A new hypothesis of dinosaur relationships and early dinosaur evolution. Nature. 2017;543: 501–506. 10.1038/nature21700 28332513

[56] Weishampel DB. Hadrosaurid jaw mechanics. Acta Palaeontol Pol. 1983;28: 271–280.

[57] Norman DB, Sues H-D, Witmer LM, Coria RA. Basal Ornithopoda The Dinosauria. Second Berkeley, California: University of California Press; 2004 pp. 393–412.

